# Comparative Compressibility of Smectite Group under Anhydrous and Hydrous Environments

**DOI:** 10.3390/ma13173784

**Published:** 2020-08-27

**Authors:** Yongmoon Lee, Pyosang Kim, Hyeonsu Kim, Donghoon Seoung

**Affiliations:** 1 Department of Geological Sciences, Pusan National University, Busan 46241, Korea; lym1229@pusan.ac.kr; 2 Department of Earth Systems and Environmental Sciences, Chonnam National University, Gwangju 61186, Korea; 197944@jnu.ac.kr (P.K.); 197942@jnu.ac.kr (H.K.)

**Keywords:** high-pressure, smectite, bulk moduli, anhydrous and hydrous environments, synchrotron X-ray powder diffraction, pressure-transmitting media

## Abstract

High-pressure synchrotron X-ray powder diffraction studies of smectite group minerals (beidellite, montmorillonite, and nontronite) reveal comparative volumetric changes in the presence of different fluids, as pressure transmitting media (PTM) of silicone oil and distilled water for anhydrous and hydrous environments at room temperature. Using silicone oil PTM, all minerals show gradual contraction of unit-cell volumes and atomistic interplane distances. They, however, show abrupt collapse near 1.0 GPa under distilled water conditions due to hydrostatic to quasi-hydrostatic environmental changes of water PTM around samples concomitant with the transition from liquid to ICE-VI and ICE-VII. The degrees of volume contractions of beidellite, montmorillonite, and nontronite up to ca. 3 GPa are ca. 6.6%, 8.9%, and 7.5% with bulk moduli of ca. 38(1) GPa, 31(2) GPa, and 26(1) GPa under silicone oil pressure, whereas 13(1) GPa, 13(2) GPa, and 17(2) GPa, and 17(1) GPa, 20(1) GPa, and 21(1) GPa under hydrostatic and quasi-hydrostatic environments before and after 1.50 GPa, respectively.

## 1. Introduction

Smectite is the most abundant two-dimensional phyllosilicate mineral group and comprises the most common natural materials, which occur by weathering, diagenesis, and hydrothermal alteration [1,2]. It is an important material for applications such as engineered barrier systems for nuclear waste storage and industrial catalysts [3,4]. Fundamental studies on the elastic properties of the smectite group have provided thermodynamic parameters that relate to the stability of materials [1,2].

**Figure 1:**
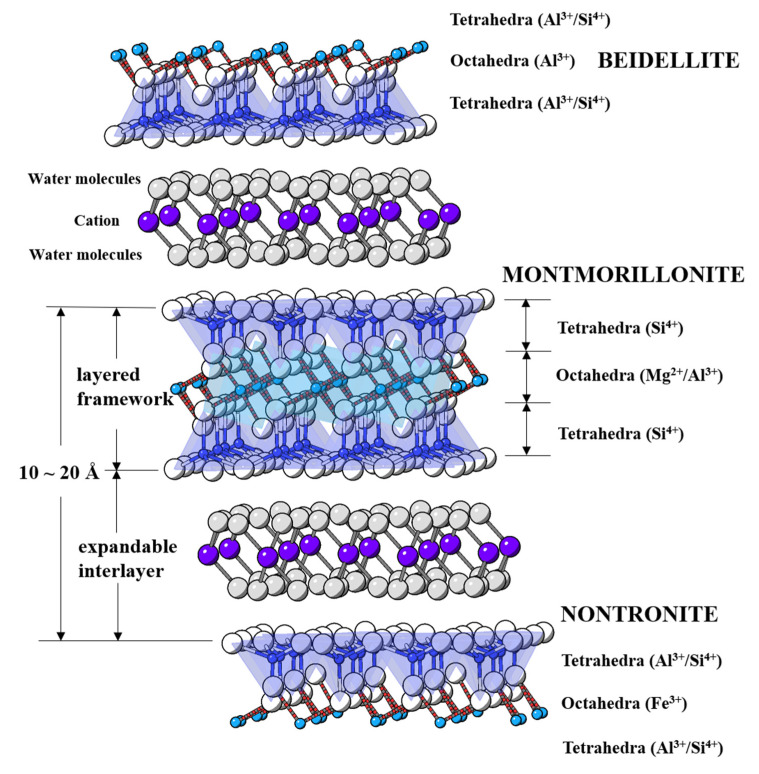
Schematic illustration of smectite structures represented by beidellite, montmorillonite, and nontronite. Blue and cyan colored circles represent the central cation in tetrahedra and octahedra comprising layered framework and white circles represent framework oxygen atoms, respectively. Purple and gray colored circles illustrate counterbalance interlayer cations and water molecules, respectively.

Representative minerals in the smectite group are beidellite, montmorillonite, and nontronite stratified by 2:1 (T–O–T) layered framework and counterbalance cation surrounded by water molecules at expandable interlayers [5,6,7]. Specifically, the montmorillonite shows predominant changes of cation charge in the octahedral layer (mostly Si^4+^ at tetrahedral site; Al^3+^ and Mg^2+^ at octahedral sites), whereas beidellite (Al^3+^ in octahedral site) and nontronite (Fe^3+^ for octahedral site) show changes in the tetrahedral layer (Al^3+^ and Si^4+^ at tetrahedral sites) (Figure 1).

One of the most interesting and distinctive properties of smectite is swelling concomitant with interlayer hydration due to the influx of surrounding water molecules [8,9]. Recent studies of phyllosilicate clays under different pressure conditions reveal that the kaolinite family (Al_2_Si_2_O_5_(OH)_4_, kaolinite, and nacrite), stratified by 1:1 (T–O) layered framework, shows swelling and superhydration of the mono-water layer under water saturated environments at 5.75(1) GPa and 460(5) °C and, subsequently, transitions to a high-pressure phase (coesite, diaspore, and topaz–OH) under consecutive pressure and temperature conditions [10,11]. A synthetic Na–hectorite (Na_0.3_(Mg)_2_(Si_4_O_10_)(F)_2_-xH_2_O), 2:1 (T–O–T) layered framework, shows anomalous swelling due to pressure-induced hydration at 2.2(1) GPa concomitant with bi- to tri-water layers increment [12].

In topologically anisotropic phases such as 2-dimensional phyllosilicates, including smectite clays, pressure-dependent unit-cell axes and volume changes are related to key structural directions defined by silicate framework sheets and interlayers. The comparative high-pressure study of smectites under anhydrous and hydrous environments is therefore important to provide compressibility that can be used with thermodynamic parameters to calculate key reactions for understanding their stabilities. 

In this work, we studied the comparative compressibility of beidellite, montmorillonite, and nontronite through high-pressure synchrotron X-ray powder diffraction experiments from ambient condition up to nearly 4 GPa using diamond anvil cell (DAC) with two different pressure transmitting media (PTM) of silicone oil and distilled water for anhydrous and hydrous environments, respectively.

## 2. Materials and Methods 

Natural smectite samples (beidellite from ID, USA., SBId-1, montmorillonite (Ca-rich) from Apache County, AZ, USA., SAz-2 from the Source Clays of the Clay Mineral Society, The Clay Mineral Society, Chantilly, VA, USA and nontronite from Uley Graphite Mine, Australian Graphite Pty Ltd, Port Lincoln, Australia, NAu-1 [13]) were ground and loaded inside a sample chamber with 200 µm diameter and 100 µm thickness at the center of a stainless steel gasket between two opposed diamond culets with 500 µm diameters.

In-situ high-pressure synchrotron X-ray powder diffraction on smectite at room temperature (25 °C) was performed at beamline 3D and 5A at Pohang Light Source II (PLS-II) at Pohang Accelerator Laboratory (PAL) in Korea. At both beamlines, the primary white beam from the bending magnet was monochromatized using Si(111) crystal DCM (double crystal monochromator), and a pin-hole was used to create an approximately 100 µm beam of monochromatic X-rays with a wavelength of 0.6888 Å. MAR345 IP detectors were used at both beamlines and the wavelength of the incident beam was calibrated using a LaB6 standard (SRM660c).

A modified 4-pin type diamond anvil cell (DAC) was used for the high-pressure experiments, equipped with two type-I diamond anvils (500 µm culet diameter) and tungsten carbide supports [14]. A stainless steel foil of 200 µm thickness was pre-indented to a thickness of about 100 µm and a 200 µm hole for the sample chamber was drilled by electro-discharge machine erosion. The powder sample of smectite (beidellite, montmorillonite, and nontronite) was placed in the sample chamber hole on a gasket with several ruby chips for in-situ pressure measurements. 

Ambient pressure data of the smectite group (beidellite, montmorillonite, and nontronite) were collected on the dry powder sample for nonswelling conditions and the wet powder samples, which are saturated by water molecules for swelling environment, respectively. Subsequently, the silicone oil and distilled water were added to the sample chamber as a pressure-transmitting media, and then, the DAC was sealed to the first pressure point. The sample pressure is measured by detecting the shift in the R1 emission line of included ruby chips [15]. The samples were typically equilibrated for about 10 min. in the DAC at each measured pressure points. The pressure was increased in steps of 0.3–0.6 GPa up to 3–4 GPa. The FIT2D program (16-041) suite was used to integrate the diffraction images into diffraction patterns.

The pressure-dependent changes in the unit-cell lengths and volumes were derived from a series of whole-profile fitting procedures using the LeBail Method implemented EXPGUI program (1251) suite [16,17]. The background was fitted with a Chebyshev polynomial with 20 coefficients, and the pseudo-Voigt profile function proposed by Thompson et al. was used to model the observed Bragg reflections [18]. The derived bulk moduli from normalized volume (*V*/*V*_0_) were calculated using the third order Birch–Murnaghan equation of state, Equation (1) [19]. The value of *B*’ for bulk moduli calculation is fixed as “4” for natural materials approximation.

(1) $$P=\frac{3}{2}B_{0}\left[{\left(\frac{V_{0}}{V}\right)}^{-7/3}-{\left(\frac{V_{0}}{V}\right)}^{-5/3}\left\{1+\frac{3}{4}\left(B_{0}^{\prime }-4\right)\left[{\left(\frac{V_{0}}{V}\right)}^{-2/3}-1\right]\right\}\right]$$

## 3. Results and Discussion

**Figure 2:**
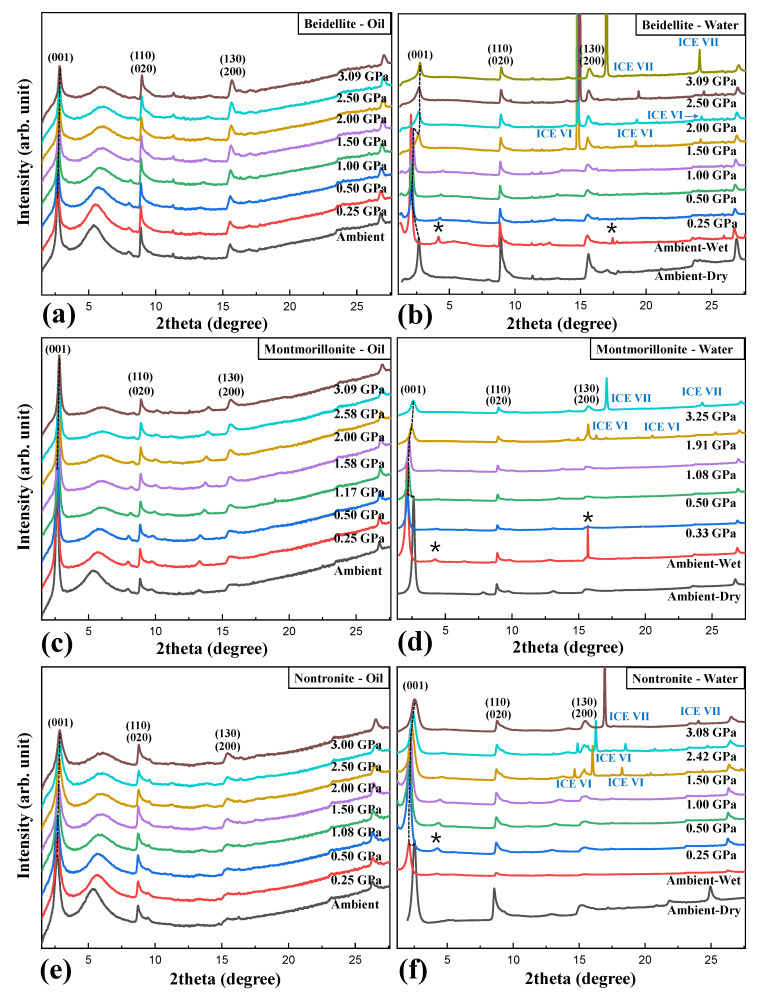
In-situ high-pressure synchrotron X-ray powder diffraction patterns of beidellite, montmorillonite, and nontronite in silicone oil and distilled water pressure transmitting media (PTM) for anhydrous and hydrous environments at room temperature. Each stacked pattern represents (**a**) beidellite oil, (**b**) beidellite water, (**c**) montmorillonite oil, (**d**) montmorillonite water, (**e**) nontronite oil, and (**f**) nontronite water, respectively. Selected Miller indices are shown. The reflections of impurities are marked as asterisks. The humps near 5.5 to 6 degrees in 2-theta in silicone oil data indicate short-range ordering of silicon oil PTM.

**Figure 3:**
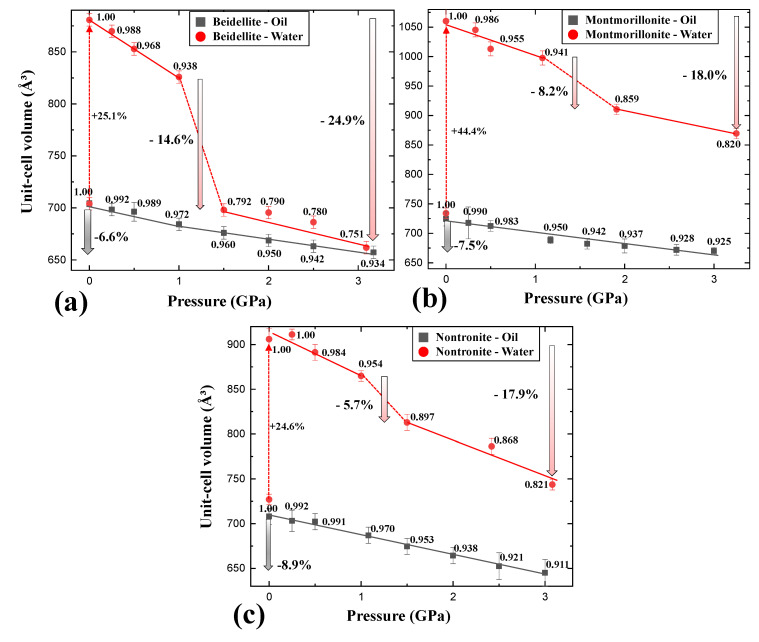
Pressure-dependent unit-cell volume changes of (**a**) beidellite, (**b**) montmorillonite, and (**c**) nontronite in silicone oil and distilled water PTMs for anhydrous and hydrous environments, respectively, at room temperature. Normalized volumes are shown on data points. The dashed and bold arrows show percent changes.

**Figure 4:**
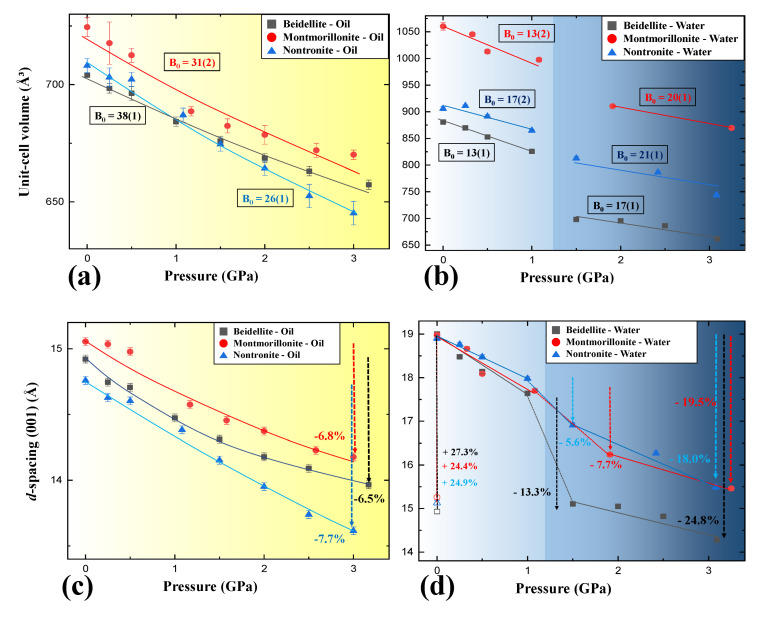
Comparative bulk-moduli of smectite under (**a**) silicone oil and (**b**) distilled water PTMs conditions at room temperature. Pressure-dependent interplane (001) distance changes of smectite under (**c**) silicone oil and (**d**) distilled water PTM conditions. The dashed arrows show percent changes.

Pressure-dependent changes in the measured in-situ synchrotron X-ray powder diffraction patterns of smectites (beidellite, montmorillonite, and nontronite) under anhydrous (silicone oil) and hydrous (distilled water) conditions are shown in Figure 2 and Figure 3 and Tables S1–S3. At ambient pressure, smectites in a water-saturated environment showed noticeable extension of *d*(001) up to ca. 19 Å, which was interpreted as interlayer expansion due to formation of tri-water layers (additional water layer intercalation), whereas *d*(001) of all samples in a silicone oil environment was constant at ca. 15 Å, which is similar length of bi-water layers (Figure 4c,d) [20]. 

**Figure 5:**
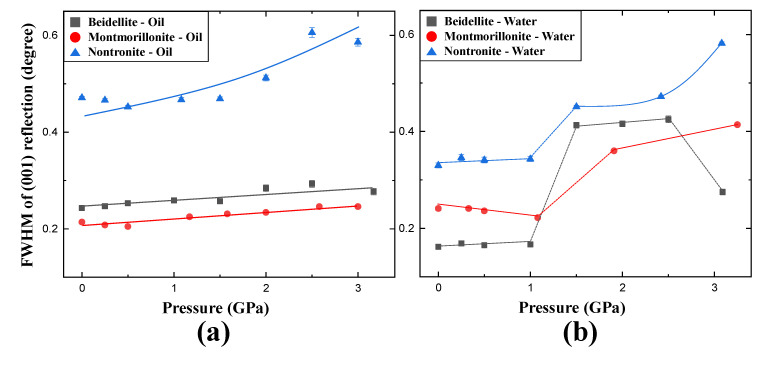
Pressure-dependent FWHM changes of the interplane (001) Bragg reflections of beidellite, montmorillonite, and nontronite under (**a**) anhydrous and (**b**) hydrous environments, respectively.

In the case of beidellite (Na_0.3_Al_2_(Si, Al)_4_O_10_(OH)_2_·nH_2_O) in the presence of silicone oil, all diffraction peaks gradually shifted to higher 2-theta showing normal compression behavior up to 3.09 GPa concomitant with contraction of unit-cell volume by 6.6% Figure 2a and Figure 3a), whereas (001) reflection shows an anomalous shift at 1.50 GPa in distilled water PTM (Figure 2b). At this pressure, the unit-cell volume of beidellite abruptly contracted ca. 14.6% and showed discontinuity of volume contraction behavior due to the changing environment around the sample from a hydrostatic to a quasi-hydrostatic condition by the transition of water PTM to ICE-VI (Figure 3a). Subsequently, the (001) reflection contracted gradually up to the final pressure of ca. 3 GPa with a volume contraction of ca. 24.9% Figure 2b and Figure 3a). Upon the change to quasi-hydrostatic condition, the FWHM (full with at half maximum) of (001) reflections also abruptly increased up to ca. 255% before and after 1.50 GPa accompanying consecutive peak shifting to a higher 2-theta angle, whereas the FWHM of (001) reflections, in case of silicone oil runs, showed gradual increments within 20% (Figure 5a,b) [21,22]. Bulk modulus, *B*_0_ (GPa), of beidellite was calculated to be 38(1) GPa under a silicone oil environment, 13(1) GPa under hydrostatic water pressure, and 17(1) GPa under quasi-hydrostatic water pressure, respectively (Figure 4a,b and Table 1).

Unit-cell volumes of montmorillonite (Ca_0.3_(Al, Mg)_2_(Si_4_O_10_)(OH)_2_·nH_2_O) and nontronite (Ca_0.5_Fe_4_(Si, Al)_4_O_10_(OH)_2_·nH_2_O) in silicone oil PTM gradually decreased with contraction of 8.9% and 7.5% up to 3.0 GPa, respectively (Figure 3b,c). Similarly to beidellite, which showed an anomalous peak shift with drastic volume contraction of 14.6% at 1.50 GPa under water saturated PTM condition, montmorillonite and nontronite showed discontinuous behaviors with 5.7% and 8.2% of drastic volume contraction near 1.5 GPa, where the hydrostatic environment started to break down and concomitant with existence of ICE-VI and VII in diffraction patterns (Figure 2 and Figure 3). Bulk moduli, *B*_0_ (GPa) of montmorillonite and nontronite was calculated to 31(2) GPa, and 26(1) GPa under silicone oil environment, and 13(2) GPa and 17(2) GPa under hydrostatic water pressure, and 20(1) GPa and 21(1) GPa under quasi-hydrostatic pressure, respectively (Figure 4a,b and Table 1).

Overall, comparative compressional behaviors under two different PTM conditions suggest that beidellite, specifically, showed relative higher incompressibility than others under silicone oil PTM conditions, whereas lower under water PTM conditions (Table 1). Under the water PTM conditions, smectite group minerals showed compressional discontinuities accompanying the “hydrostatic to quasi-hydrostatic” environmental change around the sample by the transition of water PTM to ICE-VI near 1.5 GPa. 

We, therefore, undertook the calculation of the atomistic interplane distance of (001) plane to understand the relationship between interlayer changes of smectite group minerals and different fluid conditions as a function of pressure (Figure 4c,d). In silicone oil PTMs, the *d*(001) of beidellite, montmorillonite, and nontronite up to ca. 3 GPa gradually reduced ca. 6.5%, 6.8%, and 7.7% accompanying volume contraction of ca. 6.6%, 8.9%, and 7.5%, respectively. Montmorillonite, specifically, showed ca. 2.1% greater volume contraction behavior than *d*(001) contraction. Under the water PTM conditions, smectite minerals showed similar *d*(001) distances near 19 Å in wet conditions, showing interlayer expansion due to additional intercalation of water layer inside interlayer from water-saturated environments, whereas they showed about 22% differences in maximal unit-cell volumes, respectively (Figure 4a,d). Before 1.0 GPa, the interlayer of three smectites showed similar compressional behaviors by pressure. In consecutive increasing pressures over 1.50 GPa, however, beidellite, montmorillonite, and nontronite showed ca. 13.3%, 7.7%, and 5.6% of the abrupt collapse of interlayer distances, whereas they showed 14.6%, 5.7%, and 8.2% of unit-cell volume contraction. 

## 4. Conclusions

This study establishes that beidellite, montmorillonite, and nontronite show different behaviors in anhydrous and hydrous PTM environments. All samples show modulated volume contraction by pressure and with bulk moduli of ca. 38(1) GPa, 31(2) GPa, and 26(1) GPa for beidellite, montmorillonite, and nontronite, respectively. When samples are exposed to water-saturated conditions, volume expansions are observed accompanying *d*(001) swelling behaviors up to 19 Å. In water PTM, they show bulk moduli of 13(1) GPa, 13(2) GPa, and 17(2) GPa for beidellite, montmorillonite, and nontronite, respectively, before 1.0 GPa. After 1.50 GPa, hydrous environments change to quasi-hydrostatic conditions due to the transition of liquid water PTM to ICE-VI and ICE-VII. We then observed further enhancement of bulk moduli for beidellite, montmorillonite, and nontronite to be 17(1) GPa, 20(1) GPa, and 21(1) GPa, respectively. From the results in change of bulk moduli before and after 1.50 GPa, we expect that water layers can interact with water molecules of PTM under the hydrostatic pressure conditions. After the change to quasi-hydrostatic conditions, however, the interaction might be prohibited due to the liquid-to-solid transition of water PTM and, therefore, they show normal volume contractions after 1.50 GPa. High-pressure spectroscopic investigations are underway to understand the detailed relationship between anomalous changes in bulk moduli and water interactions under hydrous and anhydrous environments.

## Figures and Tables

**Table 1:** Bulk moduli of beidellite, montmorillonite, and nontronite under silicone oil and distilled water PTMs (ESD’s (estimated standard deviations) are in parentheses).

Bulk Modulus, *B*_0_ (GPa)		Beidellite	Montmorillonite	Nontronite
Silicone-Oil		38(1)	31(2)	26(1)
distilled water	(hydrostatic)	13(1)	13(2)	17(2)
	(quasi-hydrostatic)	17(1)	20(1)	21(1)

## Supplementary Materials

The following are available online at https://www.mdpi.com/1996-1944/13/17/3784/s1. Figure S1: Pressure-dependent changes of the interplane (001) distances of beidellite, montmorillonite, and nontronite in present of silicone-oil and distilled water PTMs. Table S1: Final refined unit-cell parameters, volumes, *d-spacing* of (001) plane, and FWHM of (001) reflections of beidellite under pressure conditions, at room temperature. Table S2: Final refined unit-cell parameters, volumes, *d-spacing* of (001) plane, and FWHM of (001) reflections of montmorillonite under pressure conditions, at room temperature. Table S3: Final refined unit-cell parameters, volumes, *d-spacing* of (001) plane, and FWHM of (001) reflections of nontronite under pressure conditions, at room temperature.
