# Supplementary material for: Comparative Compressibility of Smectite Group under Anhydrous and Hydrous Environments
Source: Materials (Basel). 2020 Aug 27;13(17):3784. doi: 10.3390/ma13173784 (PMC7503564; doi:10.3390/ma13173784)
Supplement: Supplementary file 1 [file materials-13-03784-s001.pdf]

materials

Supplementary

Comparative Compressibility of Smectite Group under Anhydrous and Hydrous Environments

Yongmoon Lee [1], Pyosang Kim [2], Hyeonsu Kim [2] and Donghoon Seoung [2,*]

[1] Department of Geological Sciences, Pusan National University, Busan 46241, Korea; lym1229@pusan.ac.kr
[2] Department of Earth Systems and Environmental Sciences, Chonnam National University, Gwangju 61186, Korea; 197944@jnu.ac.kr (P.K.); 197942@jnu.ac.kr (H.K.)
* Correspondence: dseoung@jnu.ac.kr; Tel.: +82-62-530-3452

Received: 19 July 2020; Accepted: 24 August 2020; Published: date

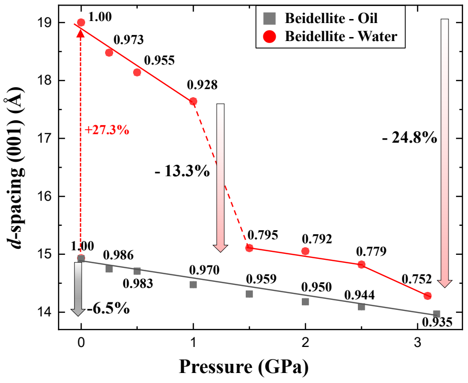

(**a**)

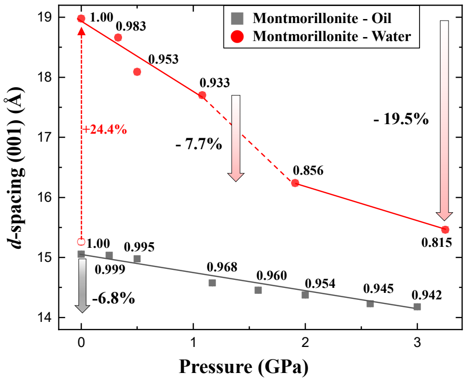

(**b**)

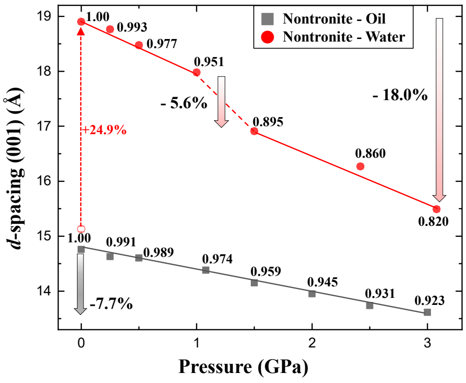

(**c**)

Figure S1. Pressure-dependent changes of the interplane (001) distances of (**a**) beidellite, (**b**) montmorillonite, and (**c**) nontronite in present of silicone-oil and distilled water PTMs for anhydrous and hydrous environments, respectively. Normalized *d-spacing* changes are shown on data points.

materials

MDPI

Table S1. Final refined unit-cell parameters, volumes, *d*-spacing of (001) plane, and FWHM of (001) reflections of beidellite under pressure conditions.[a,b,c]

PTM	Silicone-Oil						
**Pressure (GPa)**	**a (Å)**	**b (Å)**	**c (Å)**	**β (degrees)**	**Volume (Å^3)**	***d*—Spacing (001) (Å)**	**FWHM (001) Reflection (°)**
0.00	5.236(6)	9.01(2)	15.07(3)	98.0(2)	704(1)	14.92(3)	0.243(2)
0.25	5.251(8)	9.02(2)	14.87(3)	97.5(3)	698(2)	14.75(3)	0.247(3)
0.50	5.25(1)	9.03(3)	14.84(4)	97.6(3)	696(3)	14.71(3)	0.253(4)
1.00	5.237(7)	9.03(2)	14.61(3)	97.7(2)	684(2)	14.47(3)	0.259(5)
1.50	5.23(1)	9.03(2)	14.44(3)	97.7(2)	676(2)	14.31(3)	0.257(5)
2.00	5.238(8)	9.00(2)	14.30(3)	97.3(2)	669(2)	14.18(3)	0.284(7)
2.50	5.235(7)	8.99(2)	14.20(4)	97.2(2)	663(2)	14.03(3)	0.293(7)
3.17	5.229(7)	9.00(2)	14.08(4)	97.2(2)	657(2)	13.97(3)	0.277(7)
**PTM**	**Distilled Water**						
**Pressure (GPa)**	**a (Å)**	**b (Å)**	**c (Å)**	**β (degrees)**	**Volume (Å^3)**	***d*—Spacing (001) (Å)**	**FWHM (001) Reflection (°)**
0.00-Dry	5.235(6)	9.01(2)	15.08(2)	98.0(3)	704(2)	14.93(5)	0.242(5)
0.00-Wet	5.185(10)	8.94(1)	19.30(3)	100.1(3)	881(2)	19.00(5)	0.162(3)
0.25	5.227(7)	9.01(2)	18.78(3)	100.2(2)	870(2)	18.48(5)	0.169(3)
0.50	5.225(7)	8.998(7)	18.43(3)	100.2(3)	853(2)	18.14(5)	0.165(2)
1.00	5.215(6)	8.977(7)	17.92(3)	100.2(2)	826(2)	17.64(5)	0.167(2)
1.50	5.178(5)	8.923(9)	15.35(4)	100.3(4)	698(2)	15.11(5)	0.413(6)
2.00	5.182(5)	8.917(9)	15.29(4)	100.1(3)	696(2)	15.05(5)	0.416(6)
2.50	5.178(7)	8.94(2)	14.99(6)	98.6(3)	686(2)	14.82(5)	0.425(7)
3.09	5.171(5)	8.96(2)	14.47(4)	99.4(2)	662(2)	14.28(5)	0.275(6)

(a) The unit-cell parameters and volumes are derived from a series of whole profile fitting procedures using the LeBail method implemented EXPGUI program suite; (b) The interplane (001) distances and FWHM of (001) reflections were calculated using pseudo-Voigt function fitting; (c) ESD's are in parentheses.

materials

MDPI

Table S2. Final refined unit-cell parameters, volumes, *d*-spacing of (001) plane, and FWHM of (001) reflections of montmorillonite under pressure conditions.[a,b,c.]

PTM	Silicone-Oil						
**Pressure (GPa)**	**a (Å)**	**b (Å)**	**c (Å)**	**β (degrees)**	**Volume (Å³)**	***d*—Spacing (001) (Å)**	**FWHM (001) Reflection (°)**
0.00	5.27(2)	9.13(4)	15.16(2)	96.7(4)	725(4)	15.05(3)	0.214(2)
0.25	5.23(7)	9.1(2)	15.13(5)	96(1)	718(9)	15.04(3)	0.208(2)
0.50	5.28(2)	9.02(3)	15.08(2)	96.8(4)	713(3)	14.98(3)	0.205(2)
1.17	5.25(2)	9.01(2)	14.71(2)	97.8(4)	689(2)	14.58(3)	0.225(3)
1.58	5.25(2)	9.00(4)	14.57(2)	97.3(4)	682(3)	14.45(3)	0.231(3)
2.00	5.25(3)	8.99(4)	14.49(3)	97.2(7)	679(4)	14.37(3)	0.234(3)
2.58	5.26(2)	8.98(2)	14.32(3)	96.6(5)	672(3)	14.23(3)	0.246(4)
3.00	5.27(1)	8.98(2)	14.26(2)	96.3(4)	670(2)	14.18(3)	0.246(4)
**PTM**	**Distilled Water**						
**Pressure (GPa)**	**a (Å)**	**b (Å)**	**c (Å)**	**β (degrees)**	**Volume (Å³)**	***d*—Spacing (001) (Å)**	**FWHM (001) Reflection (°)**
0.00-Dry	5.25(1)	9.16(4)	15.36(2)	96.7(3)	734(3)	15.26(5)	206(2)
0.00-Wet	5.22(2)	10.71(7)	19.03(3)	94.3(4)	1060(7)	18.98(5)	0.241(4)
0.33	5.22(1)	10.72(4)	18.71(3)	94.3(2)	1045(4)	18.66(5)	0.241(4)
0.50	5.23(2)	10.70(4)	18.14(3)	94.4(4)	1013(4)	18.09(5)	0.236(4)
1.08	5.24(2)	10.76(5)	17.75(3)	94.1(3)	998(4)	17.70(5)	0.222(3)
1.91	5.23(2)	10.73(4)	16.28(4)	94.1(3)	910(3)	16.24(5)	0.360(3)
3.25	5.241(8)	10.73(4)	15.50(4)	94.0(2)	870(3)	15.46(5)	0.414(4)

(a) The unit-cell parameters and volumes are derived from a series of whole profile fitting procedures using the LeBail method implemented EXPGUI program suite; (b) The interplane (001) distances and FWHM of (001) reflections were calculated using pseudo-Voigt function fitting; (c) ESD's are in parentheses.

materials MDPI

Table S3. Final refined unit-cell parameters, volumes, *d*-spacing of (001) plane, and FWHM of (001) reflections of nontronite under pressure conditions.[a,b,c.]

PTM				Silicone-Oil			
Pressure (GPa)	a (Å)	b (Å)	c (Å)	β (degrees)	Volume (Å³)	*d*—Spacing (001) (Å)	FWHM (001) Reflection (°)
0.00	5.25(2)	9.14(2)	14.89(4)	97.6(5)	708(3)	14.76(3)	0.471(3)
0.25	5.27(2)	9.12(6)	14.76(5)	97.7(6)	703(4)	14.63(3)	0.466(3)
0.50	5.28(2)	9.12(2)	14.74(4)	97.8(4)	702(3)	14.60(3)	0.452(3)
1.08	5.25(2)	9.10(4)	14.46(4)	96.0(4)	687(3)	14.38(3)	0.467(4)
1.50	5.21(2)	9.14(4)	14.24(5)	96.4(5)	675(3)	14.15(3)	0.469(4)
2.00	5.21(1)	9.15(2)	14.04(5)	96.3(4)	664(3)	13.95(3)	0.512(6)
2.50	5.20(2)	9.14(7)	13.83(5)	96.5(6)	653(5)	13.74(3)	0.606(10)
3.00	5.20(2)	9.12(7)	13.71(7)	97.0(9)	645(5)	13.62(3)	0.586(8)
**PTM**				**Distilled Water**			
**Pressure (GPa)**	**a (Å)**	**b (Å)**	**c (Å)**	**β (degrees)**	**Volume (Å³)**	***d*—Spacing (001) (Å)**	**FWHM (001) Reflection (°)**
0.00-Dry	5.25(1)	9.14(2)	15.22(2)	96.1(4)	727(2)	15.13(5)	0.359(1)
0.00-Wet	5.28(2)	9.08(5)	19.01(4)	96.2(4)	906(4)	18.90(5)	0.330(5)
0.25	5.30(1)	9.17(2)	18.88(4)	96.3(3)	911(2)	18.76(5)	0.346(6)
0.50	5.29(2)	9.12(3)	18.59(5)	96.2(5)	891(3)	18.48(5)	0.341(6)
1.00	5.27(2)	9.13(3)	18.08(4)	96.0(3)	865(2)	17.98(5)	0.343(5)
1.50	5.28(2)	9.11(2)	17.03(4)	96.7(3)	813(3)	16.91(5)	0.451(3)
2.42	5.30(1)	9.12(2)	16.39(6)	96.7(5)	786(3)	16.27(5)	0.472(3)
3.09	5.26(2)	9.12(4)	15.59(2)	96.3(3)	744(2)	15.49(5)	0.582(3)

(a) The unit-cell parameters and volumes are derived from a series of whole profile fitting procedures using the LeBail method implemented EXPGUI program suite; (b) The interplane (001) distances and FWHM of (001) reflections were calculated using pseudo-Voigt function fitting; (c) ESD's are in parentheses.

© 2020 by the authors. Submitted for possible open access publication under the terms and conditions of the Creative Commons Attribution (CC BY) license (http://creativecommons.org/licenses/by/4.0/).
